# Impact of dietary administration of *Arthrospira platensis* free-lipid biomass on growth performance, body composition, redox status, immune responses, and some related genes of pacific whiteleg shrimp, *Litopenaeus vannamei*

**DOI:** 10.1371/journal.pone.0300748

**Published:** 2024-06-18

**Authors:** Mohamed Ashour, Mohamed M. Mabrouk, Ahmed I. A. Mansour, Ahmed F. Abdelhamid, Marwa F. AbdEl Kader, Mohamed A. Elokaby, Mohamed M. El-Nawsany, Abdelwahab A. Abdelwarith, Elsayed M. Younis, Simon J. Davies, Ehab El-Haroun, Mohammed A. E. Naiel

**Affiliations:** 1 National Institute of Oceanography and Fisheries (NIOF), Cairo, Egypt; 2 Fish Production Department, Faculty of Agriculture, Al-Azhar University, Cairo, Egypt; 3 Department of Fish Health and Management, Sakha Aquaculture Research Unit, Central Laboratory for Aquaculture Research, A.R.C, Kafrelsheikh, Egypt; 4 Department of Zoology, College of Science, King Saudi University, Riyadh, Saudi Arabia; 5 School of Natural Sciences, Ryan Institute, University of Galway, Galway, Ireland; 6 Fish Nutrition Research Laboratory, Animal Production Department, Faculty of Agriculture, Cairo University, Cairo, Egypt; 7 Department of Animal Production, Faculty of Agriculture, Zagazig University, Zagazig, Egypt; Sathyabama Institute of Science and Technology, INDIA

## Abstract

The current study aimed to assess the influence of dietary inclusion of cyanobacterium *Arthrospira platensis* NIOF17/003 as a dry material and as a free-lipid biomass (FL) on the growth performance, body composition, redox status, immune responses, and gene expression of whiteleg shrimp, *Litopenaeus vannamei* postlarvae. *L*. *vannamei* were fed five different supplemented diets; the first group was fed on an un-supplemented diet as a negative control group (C-N), the second group was fed on a commercial diet supplemented with 2% of *A*. *platensis* complete biomass as a positive control group (C-P_20_), whereas, the three remaining groups were fed on a commercial diet supplemented with graded amounts of FL at 1%, 2%, and 3% (FL_10_, FL_20_, and FL_30_, respectively). The obtained results indicated that the diet containing 1% FL significantly increased the growth performance, efficiency of consumed feed, and survival percentage of *L*. *vannamei* compared to both C-N and C-P_20_ groups. As for the carcass analysis, diets containing *A*. *platensis* or its FL at higher levels significantly increased the protein, lipid, and ash content compared to the C-N group. Moreover, the shrimp group fed on C-P_20_ and FL_10_ gave significantly stimulated higher digestive enzyme activities compared with C-N. The shrimp fed C-P_20_ or FL exhibited higher innate immune responses and promoted their redox status profile. Also, the shrimp fed a low FL levels significantly upregulated the expression of both the peroxiredoxin (*Prx*) and prophenoloxidase (*PPO1*) genes than those receiving C-N. The current results recommended that dietary supplementation with 1% FL is the most effective treatment in promoting the performance and immunity of whiteleg shrimp.

## 1. Introduction

The shrimp farming industry has expanded intensively and has become one of the most important leading global aquaculture sectors [1–3]. The Pacific whiteleg shrimp, *Litopenaeus vannamei*, has been the most widely cultivated species of all penaeid shrimp species and contributes to more than 70% of the world’s shrimp farming [4, 5]. To sustain the aquaculture industry worldwide, there are several problems to be resolved, including issues in the aquafeed industry, disease, low survivability, and poor water quality [6–9]. Moreover, the harmful effects of environmental pollution and climate change are key factors limiting the sustainability of aquaculture, fisheries, aquatic habitats, and aquatic organisms [10–12].

The shrimp feed industry has expanded by implementing a variety of strategies to deal with the global expansion in shrimp farming [7]. One of the most significant areas among these strategies is feed additive supplementation, which has become extremely important for numerous shrimp species as growth enhancers, immune stimulants, and a substitute approach for combating disease resistance [13].

The basic factors evaluating the quality of shrimp feed additives are growth performances, feed utilization indicators, biochemical composition, immune-related gene expressions, and immunological indices [14–16]. The immune system of shrimp is based primarily on innate immunity and includes cellular and humoral, enzymatic and non-enzymatic, and antioxidant effectors. Those are implemented by cellular antioxidant agents that identify invasive pathogens and activate different defense mechanisms to eliminate infections [16].

Due to its significant content of bioactive compounds, which is higher than any other sources on our planet, algal cells are still extensively used in several vital industries including aqua-feed additives [17], phytoremediation [18–21], plant growth enhancers [22], pharmaceuticals [23, 24], human food supplement [25, 26], cosmetics substances [27–29], antimicrobial activities [30, 31], bio-oil and biodiesel [21].

It is well known that *A*. *platensis* biomass contains high protein levels (up to 55–70%), crude lipids (6–11%), unsaturated fatty acids, antioxidant pigments (carotenoids), vitamins (specifically vitamin B_12_ and pro-vitamin A; β-carotene), minerals (specifically iron), and molecules that stimulate the fish feed attractability and palatability [32].

There are several forms of *A*. *platensis* supplementation in the diets of shrimp *L*. *vannamei*, such as dry powder form [33], whole-liquid extract form [34], nanoparticle form [13], derivative extract form [35] and lipid-free biomass. Each form has advantages and disadvantages. On the other hand, the procedure of addition to the diet is a key element to the success of the inclusion process. *A*. *platensis* contains up to 15.4% lipids [36]. Thus, high levels of *A*. *platensis* in the shrimp diet may result in increased excessive lipid accumulation and oxidative stress [37]. Therefore, it is essential to apply advanced procedures to eliminate the *A*. *platensis* lipid content to realize the advantages of adding *A*. *platensis* to shrimp feed on growth and health.

In our previous works, the *A*. *platensis* lipid-free biomass (FL) was successively evaluated as feed for marine rotifer, *Brachionus plicatilis*, production, and removing ammonia (phytoremediation) from aquaculture effluents [38]. The current study aims to evaluate the effect of *A*. *platensis* free-lipid biomass, FL, the biodiesel byproduct) as a dietary supplement on growth performance, feed utilization, and biochemical composition of postlarvae of whiteleg shrimp, *L*. *vannamei*, moreover, the immunological indices and immune-related gene expressions were determined.

## 2. Material and methods

### 2.1. *Arthrospira platensis* NIOF17/003

The blue-green algae, *A*. *platensis* NIOF17/003, was isolated, molecular identified, and cultured as described previously by Zaki et al. [39]. After 12 days of batch culture, under controlled culture conditions at a temperature of 28 ± 1.5°C, illumination of 3500–4500 Lux/day, continuous shaking of 80 rpm, and continuous aeration, using standard Zarrouk nutrient medium [40], the growth rate was established. The biochemical composition (at day 8^th^ of the late exponential phase) of protein (52.03% DW), lipid (8.52% DW), carbohydrates (14% DW), dry weight (0.84 DW g^−1^ L^−1^), biomass productivity (143.83 mg L^−1^ day^−1^), and lipid productivity (14.37 mg L^−1^ day^−1^) was also determined [39].

According to the worldwide standard limitations set by governing organizations (ASTM, EU), the generated biomass was evaluated as a promising source for biodiesel production, indicating its suitability to be used as a fuel. However, after lipid extraction from the *A*. *platensis* complete biomass, the *A*. *platensis* lipid-free biomass, as a biodiesel by-product, was air-dried and saved at room temperature until further applications [39]. The biochemical composition of *A*. *platensis* free-lipid biomass (FL) was determined as described previously [41].

### 2.2. Experimental procedures

#### 2.2.1. Experimental shrimp

250 postlarvae (PLs) of Pacific Whiteleg shrimp, *Litopenaeus vannamei*, were obtained from a private shrimp hatchery located in Kafr El-Sheikh City, Egypt, and gently transferred to the Fish Nutrition Laboratory, Baltim Research Station, National Institute of Oceanography and Fisheries (NIOF), Kafr El-Sheikh, Egypt. Before starting the experiment, PLs were acclimated for four weeks in Concrete tanks (5 m × 5 m × 1 m) under the same experimental controlled conditions of water temperature (26 ± 2°C), continuous aeration (dissolved oxygen, DO, 5 mg/L), and fed a commercial basal diet (45% protein) four times a day (at 7:00, 11:00, 16:00, and 21:00) until apparent satiation (Table 1). The acclimatization tanks were provided with groundwater with a salinity of 40 ppt mixed with freshwater to equalize the water entering the shrimp ponds at a salinity of 26 ± 1 ppt.

**Table 1:** The formulation and chemical composition of the basal diet (dry matter basis).

Ingredient	Experimental diet (%)
Fish meal Peru href = "#tbl1fna" a	12
Fish meal Ca Mau href = "#tbl1fnb" b	18
Wheat gluten href = "#tbl1fnc" c	3
Defatted soybean meal href = "#tbl1fnd" d	28
Squid liver powder href = "#tbl1fne" e	5
Wheat flour	25
Fish oil	3
Lecithin	1
Binder (GG) href = "#tbl1fnf" f	0.5
Cholesterol	0.1
Choline chloride 60%	0.6
MCP	0.3
Premix ^href = "#tbl1fng" g^	2.0
Ascorbic Acid	0.1
Gelatin	1.0
Lysine	0.1
Methionine	0.3
Total (%)	100
Chemical composition	%
Crude Protein	45.4
Crude lipid	7.82
Carbohydrate	33.98
Crude Fiber	3.63
Ash	9.17
Gross energy (KJ g^−1^)	19.9

^a^ Peruvian fishmeal, Pesquera Exalmar (CP 65%).

^b^ Eco-Fish Ca Mau Viet Nam (CP 60%).

^c^ VMC Group Vietnam.

^d^ Maharashtra *Solvent extraction* LTD India.

^e^ An Giang Agriculture and foods import-export joint stock company.

^f^ Binder was providing (Guar gum) was imported from Pakistan and supplied by Hoa chat Can Tho comp, Vietnam.

^g^ Premix was provided by DSM, Germany.

#### 2.2.2. Experimental diets

Five dietary treatments were tested during the trial. Each of the five shrimp groups was individually fed; an un-supplemented diet as a negative control (C-N), a commercial diet supplemented with 2% (20 g kg) of *A*. *platensis* complete biomass as a positive control group (C-P_20_), and a commercial diet supplemented with three levels of *A*. *platensis* free-lipid biomass at 1, 2, and 3% inclusion (later labeled as FL_10_, FL_20_, and FL_30_, respectively). The hypothesis based dosages of FL and/or complete biomass supplementation were carried out as indicated by Mansour et al. [42]. During the eight-week shrimp feeding trial, the diets were administered four times a day (7:00, 11:00, 16.00, and 21:00 h) at 10% of the shrimp’s total body weight as described by Sharawy et al. [13].

#### 2.2.3. Experimental culture technique and water quality

After two weeks of acclimation, 50 shrimp (0.05 ± 0.02 g) were stocked into each of 25 net hapa (0.7 × 0.7 × 1 m for each) at a total of 250 PLs for each dietary treatment. The hapa net was fixed in concrete ponds (4 × 2 × 1 m), with five hapas for each treatment. During the experiment, shrimp were kept under the experimental conditions of temperature (26 ± 2°C), salinity (26 ± 1 ppt), continuous aeration, and natural photoperiod (12:12 h dark: light). The pH (7.70 ± 0.15), NH_3_ (0.08 ± 0.01 mg L^−1^), NO_3_ (0.18 ± 0.02 mg L^−1^), and NO_2_ (0.10 ± 0.01 mg L^−1^) levels were frequently monitored [43] and confirmed to be within acceptable ranges for shrimp cultivation [44]. The nets were frequently cleaned during the experimental period, and the water turnover rate for each pond was around 10% for each pond per day by intake and output flow rates via the pond system.

### 2.3. Growth performance and feed utilization indices

The *L*. *vannamei* weights (g) were recorded at the beginning of the feeding trial (0.05 ± 0.02 g) and every 15 days afterward. At the end of the feeding trial, following a period of starvation, shrimp were counted and individually sampled for length and weight. Obtained data were used to calculate the survival rate, weight gain (WG,g), survival rate (SR,%), specific growth rate (SGR%/day), feed conversion ratio (FCR), feed efficiency ratio (FER), protein intake (PI, g), and protein efficiency ratio (PER) parameters according to the following Eqs (1–7):

WeightGain(WG,g)=Finalbodyweight(g)−Initialbodyweight(g) (1)

$$Survival\;Rate\;(SR,\%)=\frac{Total\;Final\;survived\;number\;of\;shrimp}{The\;initial\;number\;of\;shrimp}\times 100$$ (2)

$$Specific\;Growth\;Rate\;(SGR\%/day)=\frac{Ln\;Final\;body\;weight-Ln\;Initial\;body\;weight}{t}\times 100$$ (3)

$$Feed\;Conversion\;Ratio\;(FCR)=\frac{Total\;consumed\;feed\;}{WG}$$ (4)

$$Feed\;Efficiency\;Ratio\;(FER)=\frac{WG\;(g)}{Feed\;intake\;(g)}$$ (5)

ProteinIntake(PI,g)=Feedintake(g)×Protein(%) (6)

$$Protein\;Efficiency\;Ratio\;(PER)=\frac{WG\;(g)}{Protein\;intake\;(g)}$$ (7)

### 2.5. Body chemical analysis

At the end of the experiment, five shrimp from each replicate were collected to estimate the shrimp’s whole-body proximate composition. Shrimp were randomly chosen, euthanized, homogenized in a blender, oven-dried, powdered, and preserved at -20°C for further investigations. The biochemical composition percentages (crude protein, crude lipid, ash, and dry matter) were determined as previously described [41].

### 2.6. Immunological indices

From each replicate, five shrimp, following 24 hours of starvation, were randomly selected and rinsed with sterile seawater for a few seconds. Shrimp tissue samples were dissected, weighed, frozen in liquid nitrogen, and stored at– 80°C until use. For lysozyme, antioxidants, and digestive enzyme assays, the shrimp tissue samples were homogenized, after adding PBS (pH 7.4), centrifuge (20 min, 2,000–3,000 rpm), and the supernatant was carefully collected.

#### 2.6.1. Lysozyme activity assay

Serum lysozyme activity was assayed by Lysozyme (LZM) ELISA Kit (Cat NO.:SL0050FI, SunLong Biotech Co., LTD, China). During incubation of the lysozyme sample and *Micrococcus lysodeikticus* cells as the substrate, the reaction was followed by monitoring the reduction in absorbance reading at 450 nm wavelength following the manufacturer guidelines.

#### 2.6.2. Antioxidant activity assay

Serum Superoxide Dismutase (*SOD*) was colorimetrically (Cat NO.: SD2521, Biodiagnostic Co., Egypt) determined at a wavelength of 560 nm [45]. Whereas, catalase was colorimetrically (Cat NO.: CA2517, Biodiagnostic Co., Egypt) determined at a wavelength of 510 nm [46]. While, lipid peroxide (Malondialdehyde, *MDA*) was colorimetrically (Cat NO.: MD2529, Biodiagnostic Co., Egypt) determined at a wavelength of 534 nm [47].

#### 2.6.3. Digestive enzyme activity assay

The gastrointestinal tract (GIT) tissues homogenate were centrifuged and carefully separated to analyze different activities for digestive enzymes according to the manufacturing instructions. Lipase was colorimetrically (Cat NO.: 281001 Spectrum, Egy. Co. for Biotech., Egypt) determined at a wavelength of 580 nm [48], while amylase was colorimetrically (Cat. NO.: AY1050, Biodiagnostic Co., Egypt) determined at a wavelength of 660 nm [49].

### 2.7. Immune-related gene expressions

At the end of the feeding trial, three equal pools of independent samples of each five *L*. *vannamei* shrimp (whole animals) were collected, washed twice with PBS (137 mM NaCl, 2.7 mM KCl, 8 mM Na_2_HPO_4_, 1.46 mM KH_2_PO_4_, and pH 7.4), and stored in RNA later^®^ reagent (Sigma-Aldrich^®^; 1w:5v) at– 20°C as ascribed by the procedure of Goncalves et al. [50]. The total RNA extraction and quantitative real‐time PCR followed the method of Aguilera‐Rivera et al. [51]. Briefly, the TRIzol reagents protocol (TRIzol^©^; Life Technologies) was applied to extract the total amount of RNA then the obtained extraction was quantified at 260 and 280 nm using a NanoDrop spectrophotometer (Thermo Scientific). The RT2 First Strand Kit, which includes a highly successful genomic DNA removal step before reverse transcription, was used during the RNA extraction process to prevent DNA contamination. cDNA was produced in a 10‐μL estimated volume including 4 μg of the total extracted RNA, 10 × RT buffer, 10 mM dNTP, 10× random RT primers and U reverse transcriptase (Enhanced Avian RT First Strand Synthesis; Sigma‐Aldrich^©^). The first strand cDNA was generated at 59°C for 50 min. Then, the designed primers designated in this experiment, including the Peroxiredoxin (*Prx*), Prophenoloxidase (*PPO1*), p53-like protein isoform delta (*p53*), and hemocyanin subunit L5 (*L5H*) genes, were presented in Table 2 and prepared for q-RT-PCR estimation, which was conveyed into a fluorometric iQ5 thermocycler (Bio‐Rad^®^) following the Aguilera‐Rivera et al. [51] guidelines and applying the gene *β*‐actin as the housekeeping gene [52].

**Table 2:** Nucleotide primers applied to amplify the selected genes from *Litopenaeus vannamei* shrimp with quantitative real‐time-PCR.

Gene	Nucleotide primers	Accession NO.	Amplification length (bp)
Peroxiredoxin (*Prx*)	F: CATCTTCAAGGGCACTGCTG R: CGGCCTTCATTGTCTTGGAG	GQ995702	962
Prophenoloxidase (*PPO1*)	F: CCAGCAGCGTCTTCTTTACC R: GTTCAATTTCTCGCCCAGGA	AY723296	2061
p53-like protein isoform delta (*p53*)	F: CCAAGCAGCAATGTGTCAGT R: TCAGGCTGCCACTTCTTGAT	KX827274	1854
Hemocyanin subunit L5 (*L5H*)	F: ATGCTCATCGTTGAAACCCG R: TCGTGTTTTGAAATGACCTTGG	KF193065	1298
*β*-actin	F: ACTGGGACGACATGGAGAAG R: CAGGAATGAGGGCTGGAACA	JF288784	601

The applied primers of the housekeeping and target genes were designed from the conserved sequences of each gene in Genbank with Primer 5.0 software. The expression level of each gene was estimated and calculated using the ^2‐ΔΔ^C_t_ as ascribed by Livak and Schmittgen [53], where C_t_ is the value corresponding to the number of cycles in which the fluorescence was created. Each real-time PCR reaction (including cDNA synthesis) was repeated triplicate times to ensure the accuracy of the obtained results. Moreover, the qPCR values were log^2^ transformed to achieve normality and diminish data variability. Besides, the PCR efficiency for each sample was derived from the slope of the regression line fitted to a subset of baseline-corrected data points in the log-linear phase using LinRegPCR following **Ramakers et al.,** procedure [54].

### 2.8. Statistical analysis

The current feeding trial results were presented (n = 5) as the means ± standard deviation (SD). Before the data were analyzed, the normality and homogeneity assumptions were conducted and the results (%) were arc-sin transformed [55]. The statistical procedure was performed using the IBM SPSS (IBM, v.23) Statistics Software, by the one-way ANOVA followed by the Tukey’s range test, at a significant level of P ≤ 0.05. Finally, Figures were prepared by Graph Pad (Prism 8) Statistics Software [56].

## 3. Results

### 3.1. Biochemical composition of *A*. *platensis* free-lipid biomass (FL)

**Fig 1:**
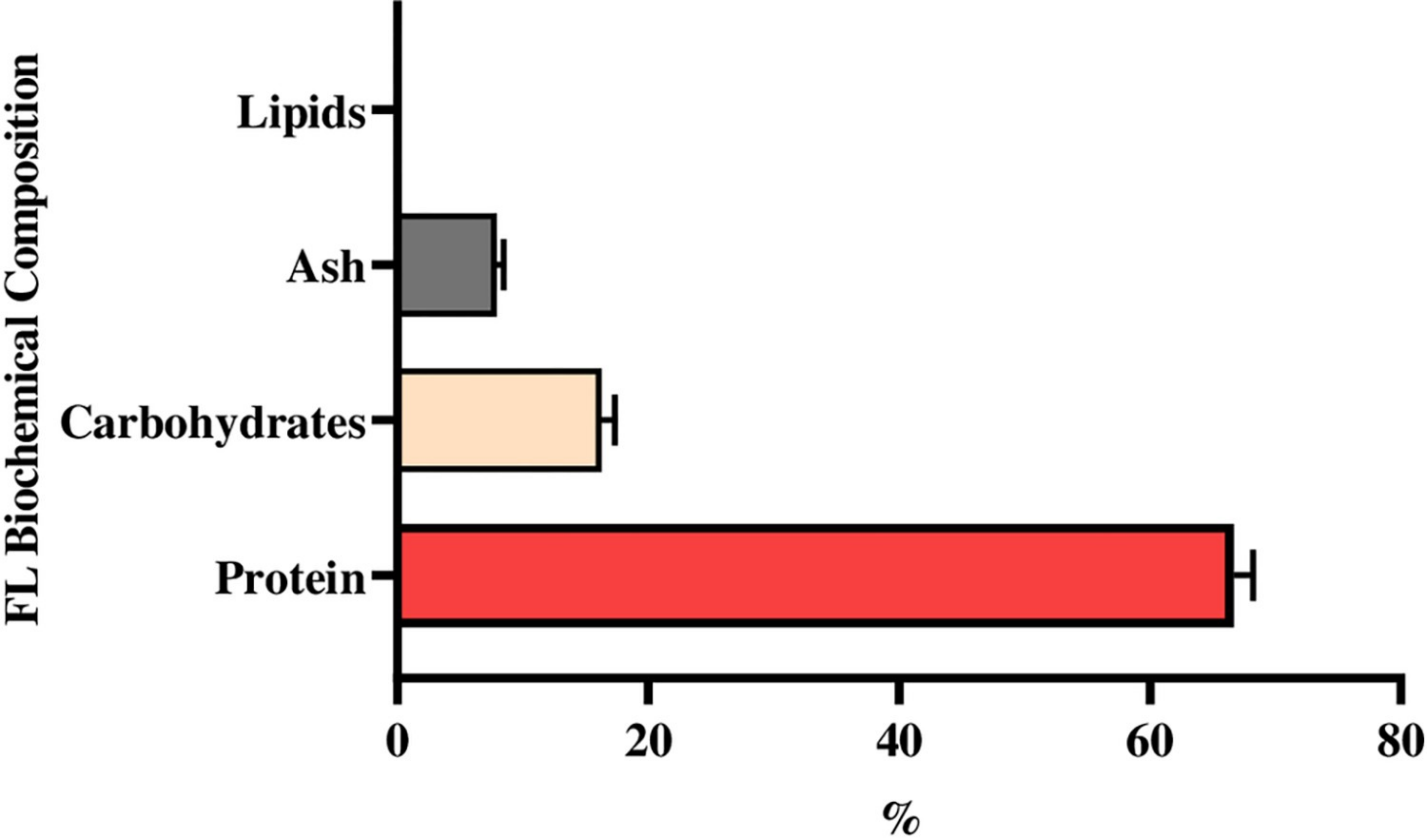
Biochemical composition of *A*. *platensis* free-lipid biomass (FL).

The chemical analyses of the free-lipid biomass of *A*. *platensis* are shown in Fig 1. Biochemical composition (% of DW) of protein, lipid, carbohydrates, and ash of *A*. *platensis* free-lipid biomass applied in the current experiment as FL (FL_10_, FL_20_, and FL_30_) was 66.7%, 0.0%, 16.29%, and 7.93%, respectively.

### 3.2. Growth performance and nutrient utilization indices

**Fig 2:**
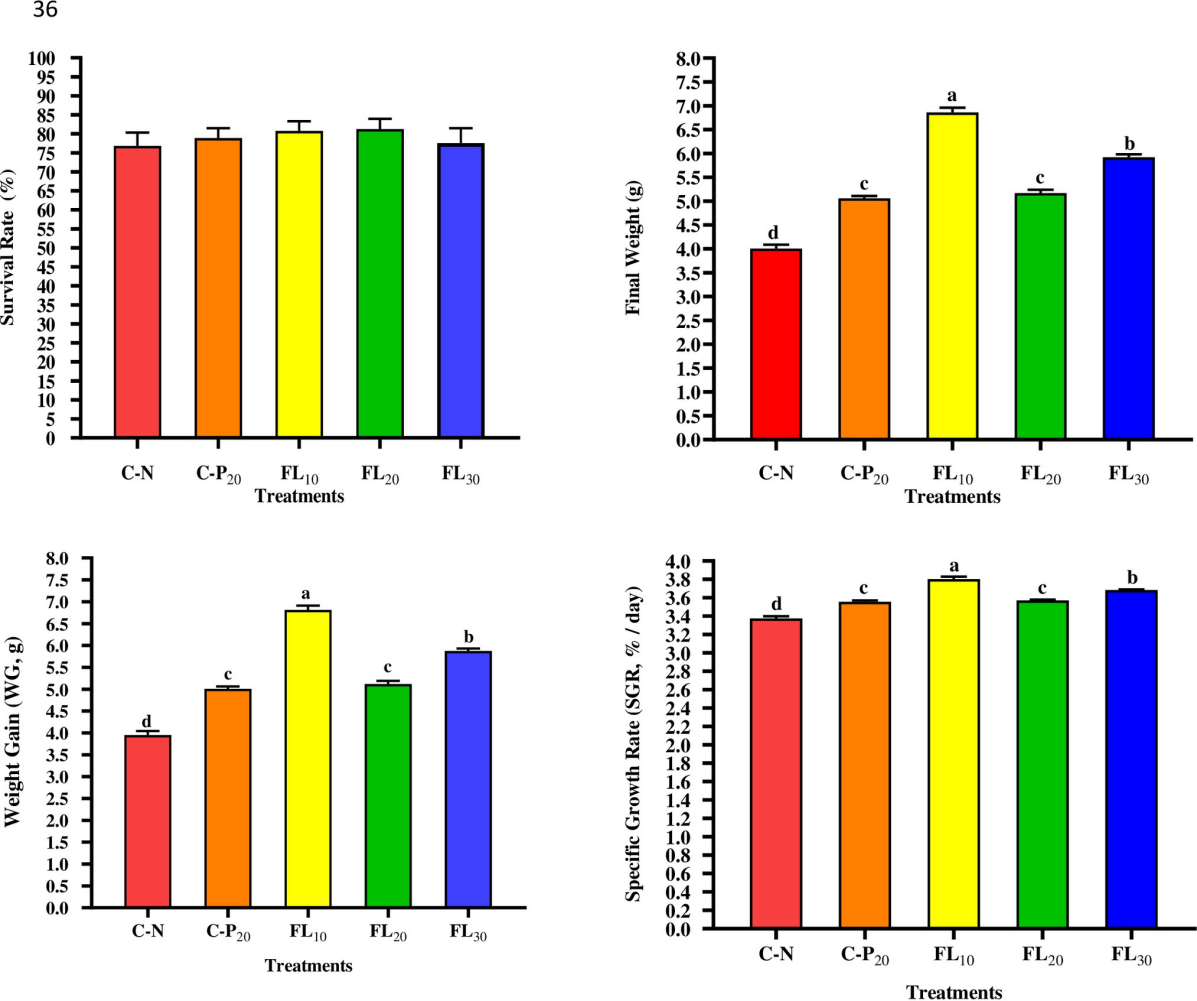
Influence of experimental diets on growth performance indices of shrimp *L*. *vannamei*. C-N: control diet (negative control), C-P_20_: control diet supplemented with 20 g kg^−1^ of *A*. *platensis* complete biomass (positive control), FL_10_, FL_20_, and FL_30_: diets supplemented with 10, 20, and 30 g kg^−1^ of *A*. *platensis* free-lipid biomass. Data were represented as means ± SD. Different letters in each column indicate significant differences (*p <* 0.05). The absence of letters in each column means that there are no significant differences.

**Fig 3:**
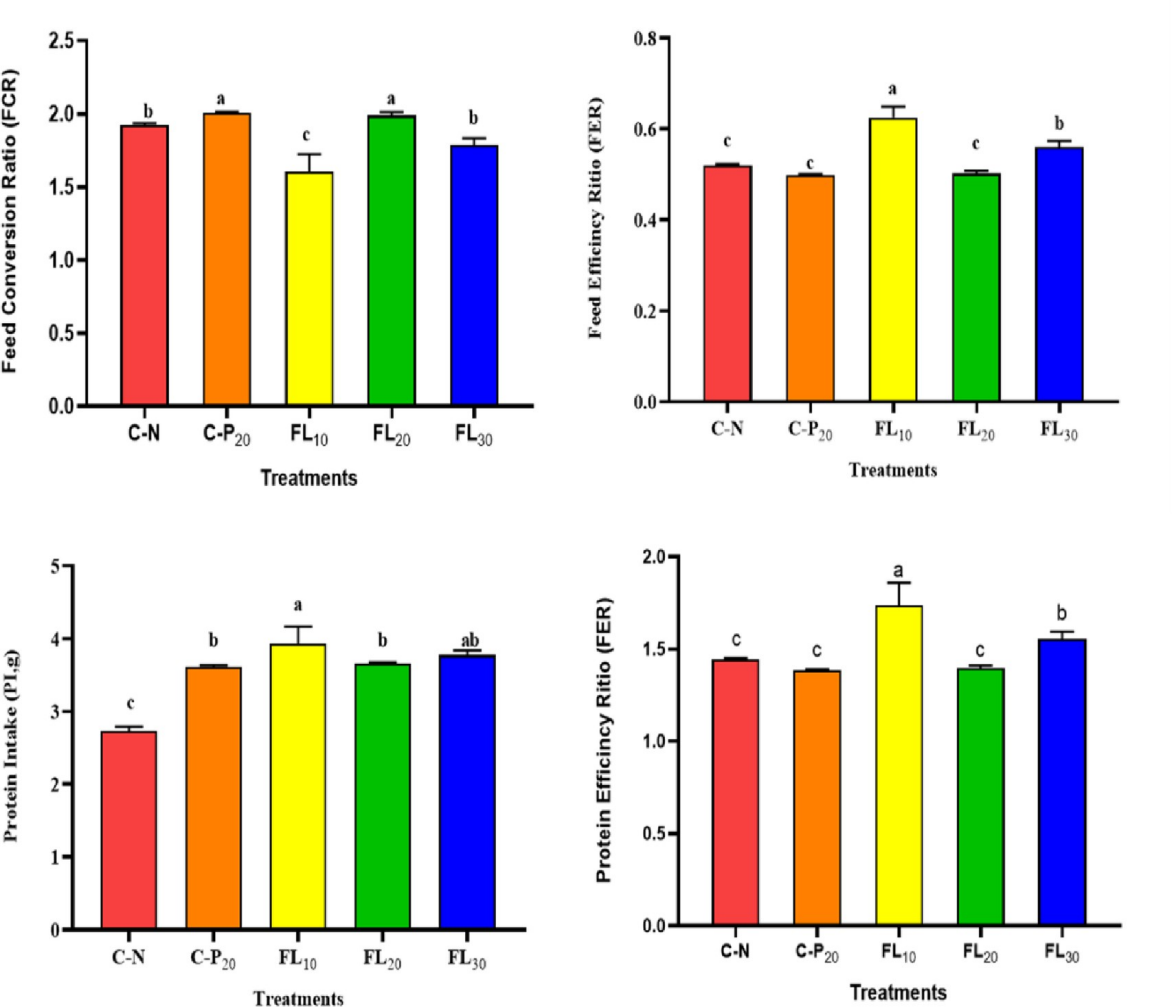
Influence of experimental diets on feed utilization indices of shrimp *L*. *vannamei*. C-N: control diet (negative control); C-P_20_: control diet supplemented with 20 g kg^−1^ of *A*. *platensis* complete biomass (positive control); FL_10_, FL_20_, and FL_30_: diets supplemented with 10, 20, and 30 g kg^−1^ of *A*. *platensis* free-lipid biomass. Data were represented as means ± SD. Different letters in each column indicate significant differences (*p <* 0.05).

Figs 2 and 3 shows the influences of dietary supplementations of *A*. *platensis* (complete dry weight or FL) on the survival, performance, and feed utilization of the juveniles of *L*. *vannamei*, respectively. No statistically significant differences (*p* ≥ 0.05) in survival rates between all treated and non-treated groups were noted (Fig 2). Overall, the 1% FL dietary supplementation significantly improved FBW, WG, SGR, FER, PER, PI, and significantly reduced FCR, compared to the shrimp group fed an un-supplemented diet and all other experimented diets (Figs 2 and 3).

### 3.3. Whole-body biochemical composition

**Fig 4:**
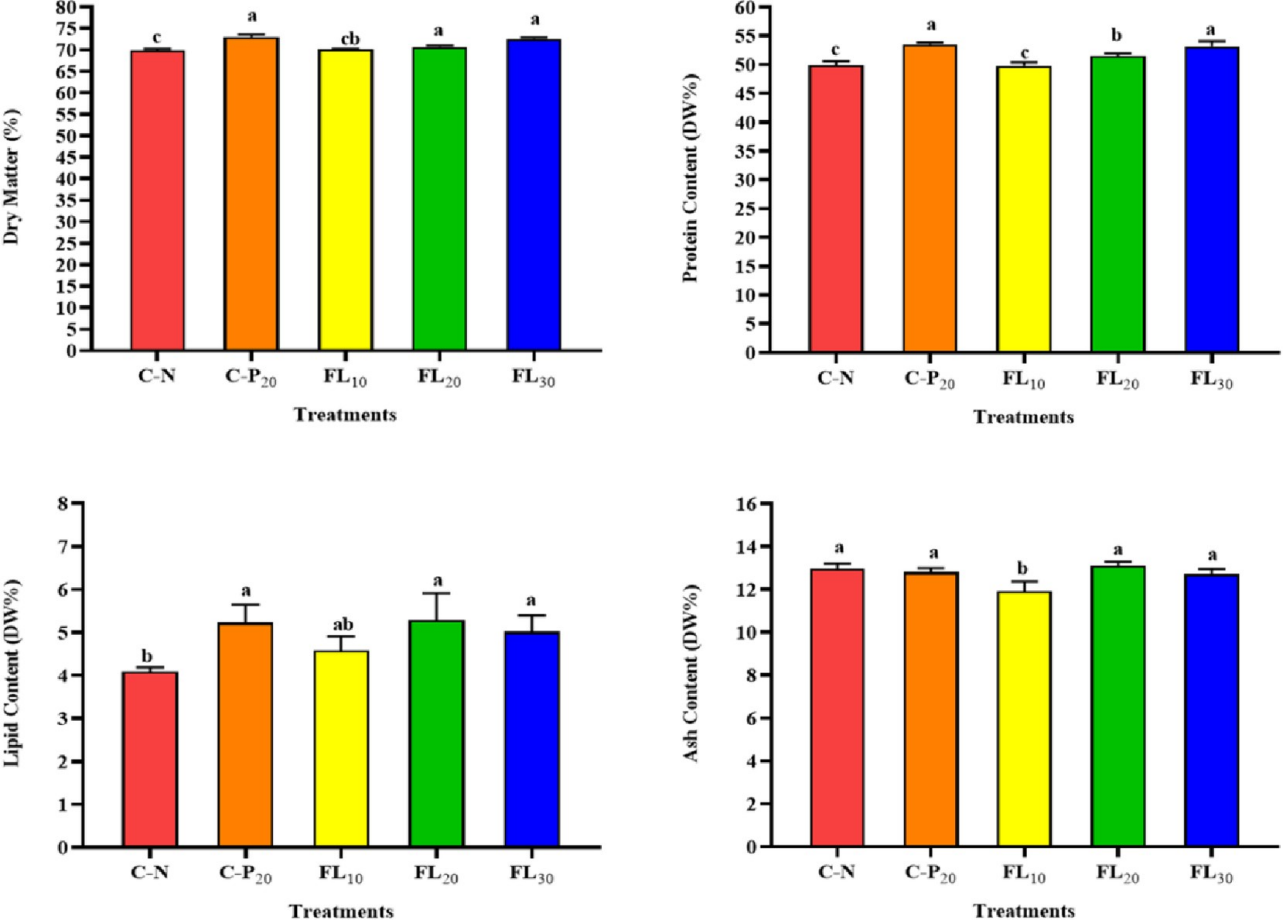
Influence of experimental diets on whole-body biochemical composition of shrimp *L*. *vannamei*. C-N: control diet (negative control); C-P_20_: control diet supplemented with 20 g kg^−1^ of *A*. *platensis* complete biomass (positive control); FL_10_, FL_20_, and FL_30_: diets supplemented with 10, 20, and 30 g kg^−1^ of *A*. *platensis* free-lipid biomass. Data were represented as means ± SD. Different letters in each column indicate significant differences (*p <* 0.05).

Fig 4 shows the whole-body biochemical composition of the shrimp fed the different dietary treatments. All shrimp treated with C-P_20_, FL_20_, and FL_30_ showed significantly (*p* ≥ 0.05) higher dry matter and lipid percentage than the C-N group and FL_10_. The lowest shrimp ash content was observed in shrimp treated with FL_10_. The highest shrimp body protein percentage was observed in the group fed C-P_20_ and FL_30_ compared with the C-N and all other treated groups of FL_10_ and FL_20_ (Fig 4).

### 3.4 immunological responses, redox status, and digestive enzyme activities

**Fig 5:**
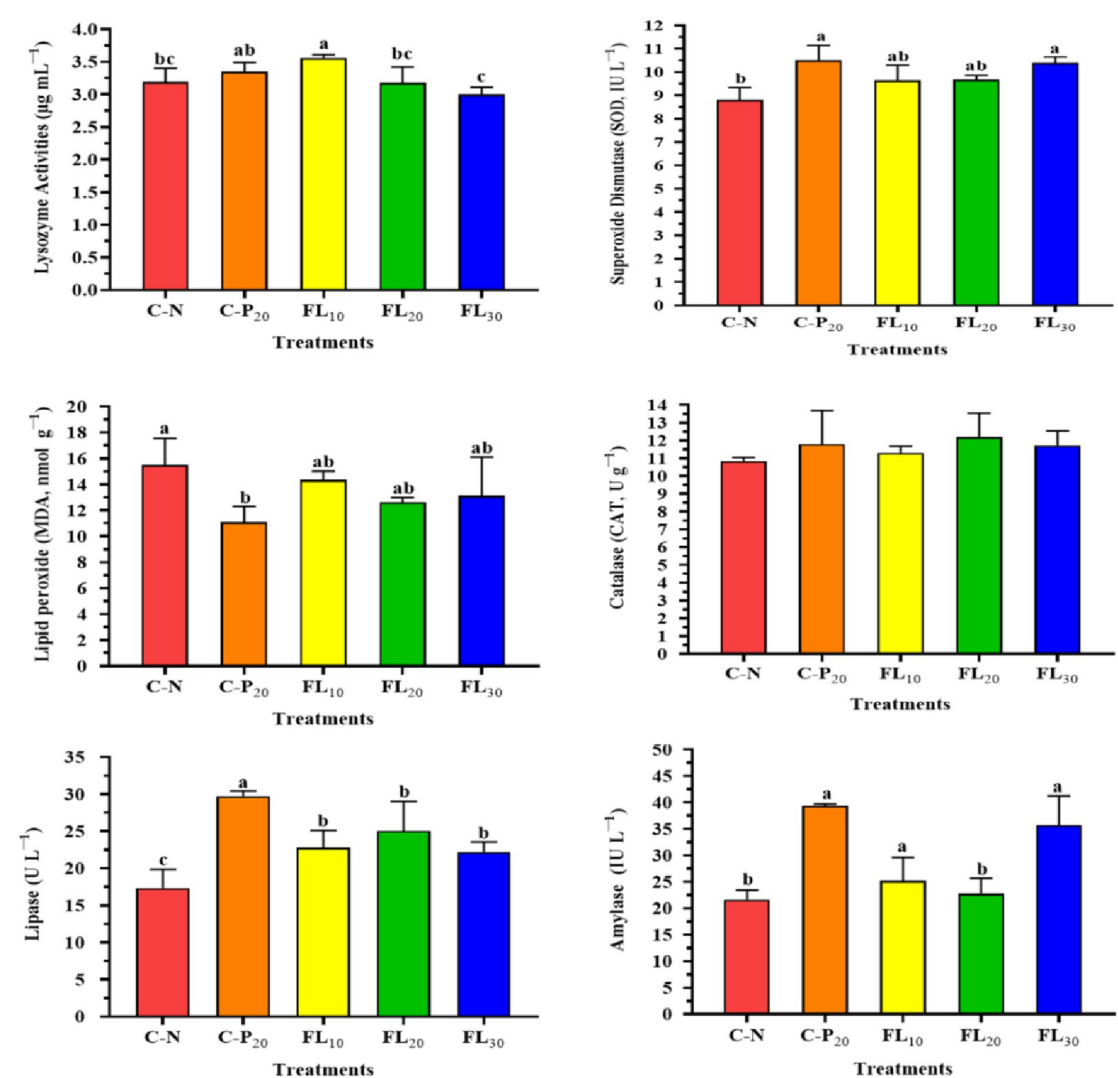
Influences of experimental diets on immunological indices of shrimp *L*. *vannamei*. C-N: control diet (negative control); C-P_20_: control diet supplemented with 20 g kg^−1^ of *A*. *platensis* complete biomass (positive control); FL_10_, FL_20_, and FL_30_: diets supplemented with 10, 20, and 30 g kg^−1^ of *A*. *platensis* free-lipid biomass. Data were represented as means ± SD. Different letters in each column indicate significant differences (*p <* 0.05). The absence of letters in each column means that there are no significant differences.

Fig 5 illustrates the influences of experimental diets supplemented with graded amounts of complete or free lipids of *A*. *platensis* biomass on immunological responses, redox status, and digestive enzyme secretions of *L*. *vannamei*. The innate immune response results showed that there were significant differences (*p <* 0.05) in lysozyme activities of shrimp groups fed diets supplemented with *A*. *platensis* (C-P_20,_ FL_10_, FL_20_, and FL_30_) compared to the C-N group. The highest shrimp lysozyme activity (3.65 μg mL^−1^) was recorded in the shrimp group treated with a low level of FL (FL_10_) (Fig 5). Shrimp provided with a complete or free lipid of *A*. *platensis* biomass-supplemented diet had significantly higher superoxide-dismutase (*SOD*) levels than shrimp fed a free basal diet (Fig 5). The highest levels of *SOD* were found in the C-P_20_ and FL_30_ groups, followed by the FL_10_ and FL_20_ groups, respectively. The effects of a supplemented shrimp diet with the complete or free lipid of *A*. *platensis* biomass on malonaldehyde (*MDA*) were significantly lower when compared to the C-N group (Fig 5). Specifically, the shrimp group fed complete *A*. *platensis* biomass exhibited the lowest *MDA* level. In contrast, there were no significant differences in catalase activity between all shrimp experimental groups. At the end of the experiment, all experimental treatments substantially altered digestive enzyme activity (lipase and amylase) (Fig 5). Shrimps fed diets containing complete or free lipid *A*. *platensis* had higher levels of amylase and lipase than shrimps fed diets without *A*. *platensis* supplementation. Specifically, shrimps fed the C-P_20_ diet had the highest levels of digestive enzymes, lipase, and amylase levels gradually increased with increasing FL content in the diets but remained lower than in the shrimp group fed the C-P_20_ diet (Fig 5).

### 3.5. Immune-related gene expression

**Fig 6:**
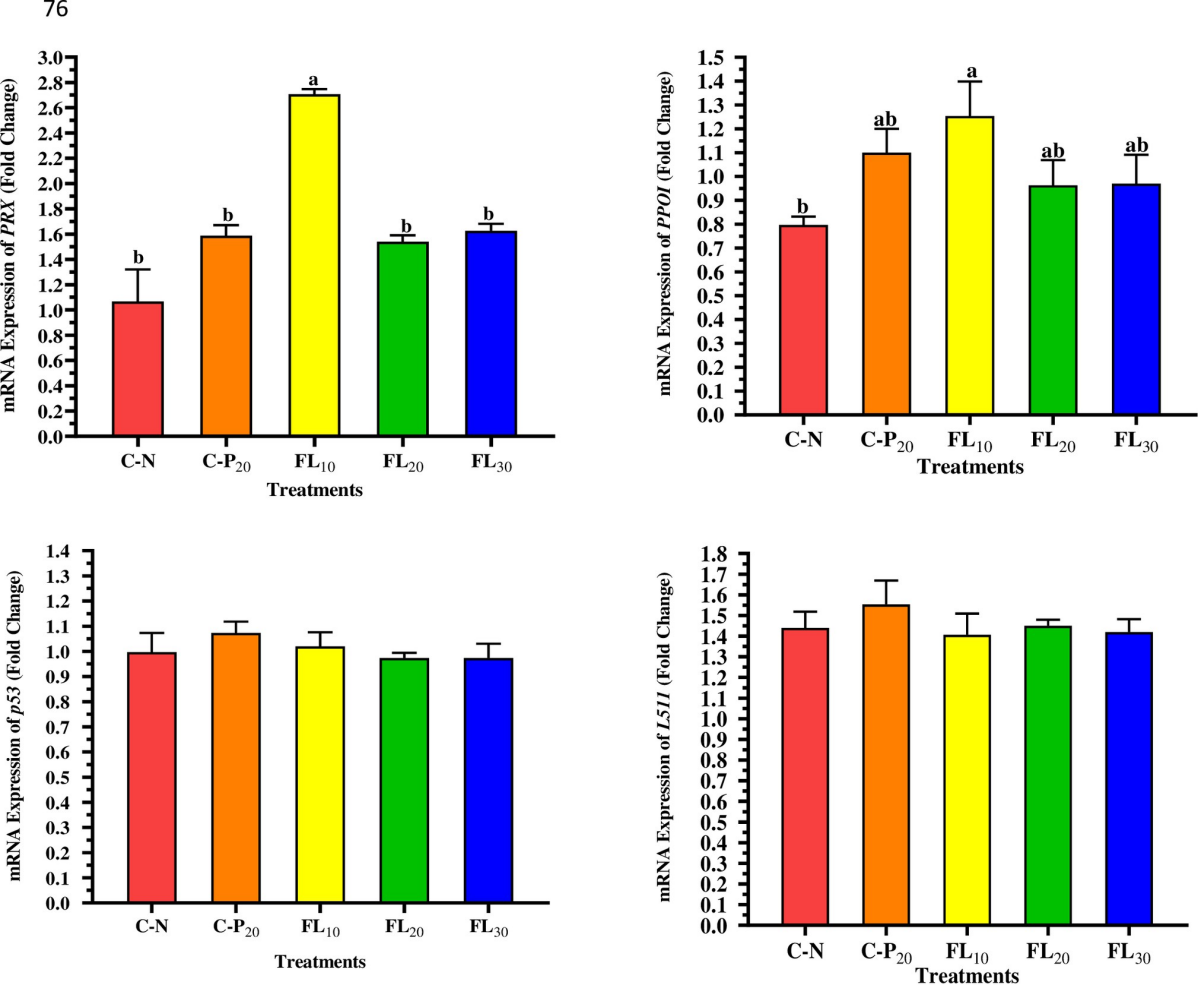
Influence of experimental diets on mRNA expression of shrimp *L*. *vannamei*. C-N: Control diet (negative control); C-P_20_: control diet supplemented with 20 g kg^−1^ of *A*. *platensis* complete biomass (positive control); FL_10_, FL_20_, and FL_30_: diets supplemented with 10, 20, and 30 g kg^−1^ of *A*. *platensis* free-lipid biomass. Data were represented as means ± SD. Different letters in each column indicate significant differences (*p <* 0.05). The absence of letters in each column means that there are no significant differences.

Fig 6 shows that the mRNA expression of *Prx* and *PPO1* genes was significantly (*p <* 0.05) influenced by dietary treatments, except for *p53* and *L5H* genes, which were not significantly altered. The expression of the *Prx* gene was highly upregulated in the treated group given an FL_10-_based diet, while FL_20_, FL_30,_ and C-P_20_ insignificantly affected the study gene when compared to the shrimp group fed diets without *A*. *platensis* addition (C-N). In contrast, feeding shrimp with two forms of *A*. *platensis* based diets significantly increased the expression level of the *PPO1* when compared to shrimp fed just a free SP diet. The shrimp fed FL_10_ (a low amount of *A*. *platensis* free lipid biomass) exhibited the highest expression of the *PPO1* gene, followed by the C-P_20_, FL_20_, and FL_30_ groups.

## 4. Discussion

Over the past decade, numerous researchers have investigated the benefits of using microalgae in whiteleg shrimp cultivation [37, 57, 58]. Although replacing fishmeal with alternative lower-cost protein sources has been examined [59, 60], using biofuel production by-products as a protein and carbohydrate source or even as feed additives for *L*. *vannamei*, has not been extensively investigated. A previous report on microalgae administration found that *A*. *platensis* blended with probiotics may promote the growth biometric indices and health status of whiteleg shrimp [61]. This current investigation was in line with our results that showed supplemented shrimp diets with varying levels of FL significantly promoted growth performance parameters, with the highest values recorded in the shrimp group fed low levels of FL (FL_10_) compared to both C-N and C-P_20_ groups.

A similar trend was observed in feed efficiency measurements, where the FL_10_ group significantly enhanced feed efficiency when compared to other treated or untreated groups. These findings imply that *A*. *platensis* or its by-product-supplied meal has high quantities of protein, which may have a favorable influence on whiteleg shrimp growth and feed efficiency indices when compared to the group fed an algal-free diet. Furthermore, our findings using *A*. *platensis* by-product are consistent with the findings of Cuzon et al. [62], who included an 8% lipid-free fraction of *A*. *platensis* meal and demonstrated higher growth and survival in *Penaeus japonicus*. In another report, Nakagawa and Gomez-DÍaz [63] reported a marked enhancement in the performance, survival percentage, pigmentation level, and protein utilization of giant freshwater shrimp (*Macrobrachium rosenbergii*) fed diets supplementing with 5–10% whole *A*. *platensis* meal and attributed the improvements to protein assimilation promotion. In addition, the inclusion of 9% defatted microalgae *Nannochloropsis* or *Thalassiosira weissflogii* meals resulted in higher growth performance of *L*. *vannamei* [64].

Lipid free algae would have a higher concentration of protein leading to less interference in terms of digestion and assimilation of released amino acids. This is a likley explanation for the superior performance of the FL fed groups over the other treatments evaluated. It is known that shrimp *L*. *vannamei* have an inferior ability to procees fats and oils in the diet due to a reduced emusfication capacity of the hepatopancreas compared to fish [65]. Most aquatic species have an advanced biliary circulation and entero-hepatic system with the release of bile salts, unlike shrimp. Lipid digestion in shrimp is mainly an intracellular activity in the hepatopancreas epithelium, from which lipids are conveyed to the target tissues and organs by the haemolymph as carrier lipoproteins. The formation and absorption of lipid micelles from the lumen of the hepatopancreas tubuli is therefore a constraint in the lipid digestion and assimilation process[66].

Recently, Namaei Kohal et al. [67] reported that most growth indicators, including final weight, specific growth rate, and average daily growth rate, were considerably higher in red cherry shrimp (*Neocaridina davidi*) fed diets supplemented with 10% *Arthrospira platensis*. The improvements in growth and efficiency of consumed diet in shrimp fed 1% FL were shown to be connected to that of a meal supplemented with microalgae by-product proven to be a rich source of carotenoids [68] and was regarded as an appropriate and safe feed additive for *L*. *vannamei*. Besides, previous reports indicated that bioactive compounds (such as growth hormones, nucleotides, vitamins, and minerals, free amino acids and fatty acids, pigments, and molecules up-regulating gene expression) in diets containing *A*. *platensis* might constitute effective agents to promote the functionality of the product and also improve feed consumption by shrimp due to gustatory and olfactory properties[69, 70].

The current study reported that feeding *A*. *platensis* or higher levels of its byproducts to whiteleg shrimp stimulated the activity of digestive enzymes, including ɑ-amylase (carbohydrate digestion). However, while lipase level was significantly higher in all dietary treatments that included FL compared to the C-N group, we discovered that enzyme activity peaked at the medium level of FL (2%), and then declined with increasing levels of FL (up to 3%) in the diet unexpectedly and inexplicably. This finding is inconsistent with reports by Namaei Kohal et al. [67]. It might be attributed to the fact that shrimp fed complete *A*. *platensis* or higher doses of FL have the potential to promote the recycling process, which is thought to be a result of both compartmentalizations produced by the presence of the peritrophic membrane and fluid movement in the midgut lumen [71]. Also, this theory predicts that an increase in protein or starch in the diet generated by *A*. *platensis* supplementation would result in the displacement of the corresponding digestive enzymes, leading to a larger recovery of these enzymes in the feces [72].

The shrimp body content in protein and lipids fed complete or by-product biomass of *A*. *platensis* increased in parallel with increasing dietary inclusion levels. The *A*. *platensis* based diet’s palatability might enhance feed intake, which subsequently increases body carcass composition [32, 73]. The observed findings were found to be consistent with Radhakrishnan et al. [73] in *M*. *rosenbergii* fed diets containing higher levels of *A*. *platensis*. Conversely, Namaei Kohal et al. [67] demonstrated that the protein content increased with dietary *A*. *platensis* levels up to 10%, but fat content was reduced with rising Spirulina levels in the caridean red cherry shrimp (*Neocaridina davidi*). Whereas, Namaei Kohal et al. [67] demonstrated that protein content increased with dietary *A*. *platensis* levels up to 10%, but fat content reduced with rising *A*. *platensis* levels in the caridean red cherry shrimp *N*. *davidi*. The difference in our study results and other research findings may be attributed to the applied microalgal species and their protein and fat content, the shrimp species, the application technique, and diet palatability.

As a crustacean, shrimp lack adaptive immunity, hence their health is mostly dependent on non-specific immune functions [74]. Superoxidase dismutase (*SOD*) and lysozyme are enzymes that neutralized cellular free radicals and collapse pathogenic bacterial cell walls, respectively [75]. Furthermore, an increase in *MDA* levels indicates an increase in free radical production, hence it is widely applied as a biomarker of oxidative stress [76]. When compared to the C-N group, shrimp fed the complete or by-product of *A*. *platensis* had higher *SOD* activity. These findings correlated with serum lysozyme activity, where shrimp fed low levels of *A*. *platensis* by-products (FL_10_) had much higher levels than the other enriched treatments and the C-N group. Conversely, *MDA* levels were significantly lower in all shrimp groups fed *A*. *platensis* complete biomass or by-products as compared to the C-N group. These findings are consistent with prior research that indicated shrimp fed *A*. *platensis* supplemented diets had improved non-specific immune responses, as well as enhanced redox status [70, 77, 78].

It is commonly known that in addition to phycocyanin, *A*. *platensis* or its by-products includes several bioactive molecules specifically, carotenoids and xanthophyll molecules, which have multiple double bonds that bind with free radicals and regulate inflammatory pathways [79]. Furthermore, Khan et al. [80] demonstrated that different *Spirulina* preparations alter the immune system through increasing macrophage phagocytic activity, promoting antibody and cytokine production, increasing NK cell accumulation in tissue, and activation and migration of T and B cells.

The peroxiredoxin (*PRX*) class of proteins are thiol-specific antioxidants found in all eukaryotes and prokaryotes [81]. These proteins serve a critical role in protecting shrimp against oxidative stressors when exposed to physical, chemical, or biological stress, which causes acute oxygen deprivation and irregular metabolic pathways, leading to the production of excessive quantities of free radicals [82]. Moreover, the transcription of the *PPO1* gene in *L*. *vannamei* was discovered to be associated with the maturation of crystal cells, which contain the enzymes required for humoral melanization, which would be linked with a variety of immunological responses [83]. In terms of the underlying mechanisms by which *A*. *platensis* has a beneficial action on shrimp, it was observed that shrimp hemocytes incubated in Spirulina dried powder (1 mg per mL) activated innate immunity, as evidenced by the recognition and binding of a recombinant protein of lipopolysaccharide and 1,3-*β*-glucan binding protein (*LGBP*), degranulation of haemocytes, a reduction in the percentage of large cells, increases in phenoloxidase (*PO*) and serine proteinase activities, activated superoxide anion levels, and up-regulated *LGBP* gene transcript [77]. In this experiment, shrimp-fed diets containing a low level of LF (10 g kg^-1^ diet) exhibited significant upregulation of both the *Prx* and *PPO1* gene transcripts when compared to other groups. Thus, more research is required to determine if dietary FL may substantially stimulate immunological or antioxidant-related metabolites in *L*. *vannamei*.

## 5. Conclusion

Several forms of microalga *A*. *platensis* supplementation, and/or their extracts, have been applied in the diets of shrimp *L*. *vanname*. However, this study revealed that free lipid biomass from *A*. *platensis*, compared to the whole-dry weight form, might improve whiteleg shrimp performance, chemical body composition, antioxidant activity, and immunological responses. Diets supplemented with a 10 g/kg diet of *A*. *platensis* free-lipid-biomass had the higher shrimp growth rate and superior feed efficiency, moreover, they also achieved the largest improvement on the innate immune response.
